# Paramedic experiences of using an enhanced stroke assessment during a cluster randomised trial: a qualitative thematic analysis

**DOI:** 10.1136/emermed-2019-209392

**Published:** 2020-06-16

**Authors:** Joanne Lally, Anu Vaittinen, Graham McClelland, Christopher I Price, Lisa Shaw, Gary A Ford, Darren Flynn, Catherine Exley

**Affiliations:** 1 Population Health Sciences Institute, Newcastle University, Newcastle upon Tyne, Tyne and Wear, UK; 2 Research and Development, North East Ambulance Service NHS Foundation Trust, Newcastle upon Tyne, Tyne and Wear, UK; 3 Stroke Research Group, Population Health Sciences Institute, Newcastle University, Newcastle upon Tyne, Tyne and Wear, UK; 4 Medical Sciences Division, University of Oxford, and Oxford University Hospitals NHS Foundation Trust, Oxford, Oxfordshire, UK

**Keywords:** paramedics, qualitative research, stroke

## Abstract

**Background:**

Intravenous thrombolysis is a key element of emergency treatment for acute ischaemic stroke, but hospital service delivery is variable. The Paramedic Acute Stroke Treatment Assessment (PASTA) multicentre cluster randomised controlled trial evaluated whether an enhanced paramedic-initiated stroke assessment pathway could improve thrombolysis volume. This paper reports the findings of a parallel process evaluation which explored intervention paramedics’ experience of delivering the enhanced assessment.

**Methods:**

Interviewees were recruited from 453 trained intervention paramedics across three UK ambulance services hosting the trial: North East, North West and Welsh Ambulance Services. A semistructured interview guide aimed to (1) explore the stroke-specific assessment and handover procedures which were part of the PASTA pathway and (2) enable paramedics to share relevant views about expanding their role and any barriers/enablers they encountered. Interviews were audiorecorded, transcribed verbatim and analysed following the principles of the constant comparative method.

**Results:**

Twenty-six interviews were conducted (11 North East, 10 North West and 5 Wales). Iterative data analysis identified four key themes, which reflected paramedics’ experiences at different stages of the care pathway: (1) Enhanced assessment at scene: paramedics felt this improved their skillset and confidence. (2) Prealert to hospital: a mixed experience dependent on receiving hospital staff. (3) Handover to hospital team: standardisation of format was viewed as the primary benefit of the PASTA pathway. (4) Assisting in hospital and feedback: due to professional boundaries, paramedics found these aspects harder to achieve, although feedback from the clinical team was valued when available.

**Conclusion:**

Paramedics believed that the PASTA pathway enhanced their skills and the emergency care of stroke patients, but a continuing clinical role postadmission was challenging. Future studies should consider whether interdisciplinary training is needed to enable more radical extension of professional boundaries for paramedics.

**Key messages**

**What is already known on this subject**

- Intravenous thrombolysis for acute ischaemic stroke requires a coordinated emergency response shared between ambulance and hospital services.
- Prehospital pathways promote treatment delivery but the impact of an advanced paramedic role has not been studied.

**What this study adds**

- This study was a qualitative exploration of paramedic views about the use of an enhanced stroke assessment which aimed to improve thrombolysis volume.
- Paramedics valued conducting a more detailed on scene assessment and a structured handover which subsequently promoted efficient transfer of clinical information relevant for thrombolysis.
- Paramedics found extension of their role to help with practical tasks in hospital as harder to achieve due to professional boundaries and expectations.

## Introduction

Intravenous thrombolysis using recombinant tissue Plasminogen Activator is a key element of emergency treatment for acute ischaemic stroke. Treatment requires administration within 4.5 hours of symptom onset because effectiveness is extremely time dependent.^1^ However, there is wide variability between services in the volume and speed of thrombolysis treatment and performance is suboptimal in many settings.^2^ In ambulance services, improvements have been described after raising the response priority level^3^ for suspected stroke and implementing routine hospital prenotification,^4^ but the clinical role of paramedics has remained focused on initial identification of symptoms and establishing the onset time.

The Paramedic Acute Stroke Treatment Assessment (PASTA) trial was a multisite pragmatic cluster randomised controlled trial which evaluated whether an enhanced paramedic-initiated ambulance care pathway (the PASTA pathway) could improve thrombolysis treatment volume.^5^ Paramedics were prerandomised to deliver the PASTA pathway or to continue with standard care when responding to a patient with symptoms of suspected stroke which commenced within 4 hours. The primary outcome was the proportion of patients receiving thrombolysis. Secondary outcomes included key ambulance and hospital care time intervals, and health at day 90 after stroke. The trial results are reported elsewhere.^6^ A qualitative parallel process evaluation was conducted alongside the trial to explore patients, hospital clinicians and paramedic experiences and views about the PASTA pathway. As providers of the intervention, this paper reports the paramedic findings in detail because their perspective would be the most valuable for both emergency stroke care and wider ambulance activity. Views obtained from patients and hospital clinicians will be reported separately.

## Methods

### The PASTA pathway

The PASTA pathway was activated by trial intervention paramedics if their routine clinical assessment led to an impression that acute stroke was responsible for a patient’s symptoms. Additional information was collected at scene which would be relevant to a thrombolysis treatment decision (eg, information about visuospatial and speech impairment, recent surgery and anticoagulant medication). The destination hospital was prealerted. On arrival at hospital, a structured handover was performed and then the paramedic worked alongside the hospital team to assist with initial simple care activities for up to 15 min (eg, transporting them to the radiology department, cannulation and weighing the patient). Prior to departure, the paramedic completed a checklist to confirm that the thrombolysis assessment was progressing and sought feedback from an attending hospital clinician about the stroke diagnosis and onset time.

Trial intervention paramedics had to undertake 1 hour of training and an Multiple Choice Questionnaire assessment to demonstrate retention of study information, before they were able to deliver the PASTA pathway. Hospital personnel were informed about the purpose of the trial but did not receive additional training since the research question was to consider the value of the enhanced paramedic role. As a cluster trial, patients were allocated to intervention or control groups according to whether a trained intervention or usual care paramedic was in attendance. Patient consent was obtained after arrival at hospital, there was no research consent process during the prehospital phase.

### Qualitative process evaluation: paramedic perspectives

Participants were recruited from the 453 trained intervention paramedics across the three UK ambulance services involved in the trial: North East Ambulance Service NHS Foundation Trust (NEAS), North West Ambulance Service NHS Foundation Trust (NWAS) and the Welsh Ambulance Service NHS Foundation Trust (WAST). Sampling was initially attempted on a purposive basis to ensure a range of sites, length of service and experience of the PASTA pathway. However, due to a limited response to invitations to participate, all trained paramedics were subsequently approached. Email was used to issue information and ask for expressions of interest to participate in an interview.

A semistructured interview guide was developed based on relevant literature^3 4 7–11^ and discussions with clinicians (box 1). The guide evolved iteratively during the data collection period as issues arose from earlier interviews which needed exploring subsequently during the data collection period.

Box 1 **Semistructured interview guide**

Paramedic Acute Stroke Treatment Assessment (PASTA) paramedic interview guide themes

- General paramedic background/experience.
- Experience of assessing stroke patients generally.
- Experience of PASTA pathway and routine stroke pathway.
  - On scene.
  - Prealert.
  - Handover.
  - CT scan.
  - Follow-up
- Barriers encountered.
- Training and support needs.
- Trial procedures (including consent arrangements).

Interviews aimed to (1) explore the stroke-specific assessment and handover procedures which were part of the PASTA pathway, and (2) enable paramedics to share relevant views about expanding their role and any barriers/enablers they encountered.

Interviews were conducted by JL (female, PhD) or AV (female, PhD) either face to face or by telephone. Informed consent was given by each participant, either in writing prior to the interview or verbally at the time of the interview. Verbal consent was introduced to make it easier for the mobile paramedic workforce to participate. All verbal consents were documented by the interviewers. Interviews were digitally recorded, transcribed verbatim and anonymised. Interview duration ranged from 10 to 31 min.

Data collection and analysis was an iterative process, following the combined principles of the constant comparative method^12^ and thematic analysis.^13^ The constant comparative method enabled the team to identify emerging themes from the data, which were subsequently explored in later interviews. Two researchers (JL and AV) analysed the transcripts independently and these were then compared and differences in interpretation discussed and resolved during group data sessions with the wider team. Data collection ceased when all interviews were conducted with the paramedics who came forward, and when no new themes were being generated and it appeared that data saturation had been reached. The data were managed using NVIVO qualitative data analysis software. Names and ambulance services attributed to any quotes have been removed and participants are referred to by their participant identification number. The Standards for Reporting Qualitative Research guided this manuscript.^14^

### Patient and public involvement

The work described in this manuscript was undertaken as part of a programme grant. Patient and public representatives were involved in designing the application and were part of project management groups throughout the duration of the work.

## Results

In total, 26 interviews were conducted across the three ambulance services (NEAS n=11, NWAS n=10 and WAST n=5). Participants’ length of service ranged from 14 months to 27 years with qualifications up to degree level.

Data analysis identified four key themes from the paramedics’ interview accounts, where the stages of the pathway were powerful determinants of the experiences reported by the paramedics. Therefore, the four specific stages along the clinical pathway during delivery of the PASTA pathway were the best way to structure and present the results of the thematic analysis. These are as follows:

1. Assessment by the paramedic at scene.
2. The ambulance journey and prealert to the hospital.
3. Handover to hospital clinical team.
4. Assisting in hospital and feedback on initial diagnosis by paramedic

### Assessment by the paramedic at scene

Paramedics discussed their experiences of the enhanced role and use of the PASTA pathway when conducting an on-scene assessment of suspected stroke patients. The paramedics viewed the PASTA on scene assessment process as something that brought together their existing knowledge with new skills and assessment tools into a potentially replicable process. This enabled the assessment of suspected stroke patients to proceed in a more standardised and efficient manner. Paramedics did not perceive the enhanced role in the PASTA pathway to be significantly different from their current assessment practices as a part of routine care (box 2: Quotation 1). However, they did consider the standardised format of on scene assessment along with the enhanced assessment of vision, speech and onset time beneficial to improving the efficiency and accuracy of their initial assessment at this stage of the clinical pathway.

Box 2 **Quotations from ‘assessment by the paramedic at scene’**

Q1: And to be honest, it’s [PASTA] probably stuff that we would have recorded, well I would have recorded anyway, but notnecessarily in the right order. So I liked the structured approach, and I found it easy enough. It’s no different to what we would be doing with the patient. It’s just more structured and more organised, and more reportable. (P8)

Q2: There is very little difference from how I’ve always assessed a stroke. The visual things are different, getting them to follow your finger and things like that are a bit different. I wouldn’t usually be… I would be asking people their medical history and their medication but I wouldn’t specifically be looking at whether they’re usually self-mobile and feed themselves. I wouldn’t be putting the surgical history and stuff in my handover and that. (P12)

Q3: We didn’t really do any visual disturbances or tests like cognition, and we didn’t differentiate between slurred speech and word finding difficulties. We knew it was important to figure out whether patients were on anti-coagulants or not. (P20)

Q4: I think it's probably just helped to refine it and help it a little bit more, and give that focus to have a clear direction with your questioning. But I think having actual phrases that kind of nail it down quite succinctly….The checking of peripheral vision was not something that I'd ever really specifically checked for. …. But I think being aware, now, that it can specifically alter the field of view, and they may be having difficulty with vision at the peripheries was not only enlightening, but also a useful tool to bring into the history taking and observation. (P10)

Q5: I found that, as a sort of a memory aid, a tool, it helped to focus my history. I think it does really quite effectively help bringing a paramedic on-scene time down, which at the end of the day will then result in a faster onset to CT time then, won’t it, in itself. (P7)

The new components identified within the study training included the visual assessments (peripheral vision), verbal assessments (differentiation between slurred speech and word-finding difficulty) and questions regarding recent surgery (box 2: Quotations 2 and 3). For some paramedics, these were not necessarily an entirely novel or unfamiliar part of on scene assessment, this was the case particularly for those who had recently qualified (box 2: Quotation 4). However, what was considered to be different was the way in which these components were utilised as a standardised part of assessment within the PASTA pathway.

Using a standardised format of assessment as part of their professional role was viewed positively by the participating paramedics. In addition to the perceived benefits of efficiency, speed and accuracy, paramedics considered that the standardisation of the assessment format and key questions also improved confidence in their initial diagnosis (box 2: Quotation 5).

### The ambulance journey and prealert to the hospital

It was not the intention of the PASTA pathway to change the local process for prealerting, however, having completed the enhanced on scene assessment, some paramedics reported that they attempted to provide some of the enhanced information to the receiving emergency department (ED) or stroke team during the prealert. As the PASTA intervention was driven purely by paramedics and hospital staff did not receive additional training, often ED staff receiving the prealert were unaware of the trial and were not always receptive to the more proactive paramedic role including prealert (box 3: Quotation 3). Some paramedics reported a smooth process where the receiving stroke team’s awareness and experience with the PASTA trial appeared to significantly facilitate the process (box 3, Quotations 1 and 2). This mixed experience highlighted wider variability in services and system processes, which could potentially offset some of the benefits of prehospital interventions to improve care delivery.

Box 3 **Quotations from ‘the ambulance journey and prealert to the hospital’**

Q1: Normally the hospitals, when I ring them and say that I’m part of the PASTA trial, are really, really good. Yes, I’m more confident when I ring them as well because I know more stuff now so I can answer questions easier, if that makes any sense. (P10)

Q2: I have to say, they were, on the whole, very good. I think every time I've pre-alerted, there has either been somebody from the Stroke Unit in A&E, waiting for me, or they've arrived very quickly. So that's been good. (P6)

Q3: But the trouble was that half the nurses you spoke to on the phone when you rang them to say, were just, basically they wouldn’t have a clue what you were talking about. It is becoming more apparent that there are some who do know about it. So you do get a better response from them. But some of them will try to cut you off in the middle of a sentence and say, ‘well I’m not interested in that, are they on anti-coagulants?’ Well if you let me work through it you’ll know. That’s on the pre-alert. (P18)

### Handover to hospital clinical team

Paramedics described a range of experiences of transferring a patient to the hospital ED or stroke team using the PASTA structured handover process. On the whole, the structured format of the PASTA handover was viewed as a positive addition to paramedic practice and role (box 4: Quotation 1). In particular, it was considered to be beneficial in terms of efficiency and professionalism (box 4: Quotations 2 and 3). Paramedics reported feeling more confident in their initial diagnosis and more professional in terms of the structure and reliability of the process, thus also contributing to development of their professional skillset. Overall, it was considered to provide more in-depth, quality information to the hospital team, in a standardised and more proactive manner (box 4: Quotation 4).

Box 4 **Quotations from ‘handover to hospital clinical team’**

Q1: I found quite beneficial because it’s quite structured—they found it extremely helpful, I think. Just having that in front of you, it’s much easier to fill that in on the way to hospital, and have that ready, then that gives you the structured handover, and everything's there. (P17)

Q2: When you do the handover as a PASTA handover it’s very… I don’t know the terms… I like doing it and you get all the information across, and I’ve had positive feedback every time I’ve asked about it. I feel like I give a lot of information very quickly, a lot more so than I used to when you’d pre-alert for a stroke—I think it’s a bit of both really. Because you are handing over in a specific way, like I say, in my handover I wouldn’t usually say about surgical history for a stroke but I can see why that’s relevant. Whereas before it was just purely what the symptoms are and when it started, that was it. (P12)

Q3: To be honest, from my point of view it made me feel more competent. I think it might be my imagination but I think it makes us look a little bit more professional if that makes sense. (P19)

Q4: I think the system does work well, and I think it could be applied to many things. I mean, the structured handover means that you don’t forget anything, you don’t miss anything out, and could be applied to every handover, really, in some respects. To have that structure would make it easier and actually, probably, would make it less daunting, when you’re coming to do a particular, like, the PASTA one. If you’re using that structure all the time, sort of thing, you just add the extra bits in that are relevant to stroke. So I did really like that. I think it helps. (P8)

Q5: Well as I said, the very first one they weren’t even aware of, as I say the sister had heard of it but didn’t really know anything about it. So I spent most of my time there actually explaining what it was. She said, ‘It’s good but then there’s no way they’ll ever meet the 15 meeting CT time target. Yes, so the difficulty is that you know all about the trial and you’re trying to hand it over to people that don’t necessarily know or understand the full?’ (P18)

Q6: Just having the knowledge, people not aware of it, just too busy to consider it—I think just because of the nature of things and how busy it is, there was often only a nurse to take handover, you know, not handing over to a doctor. I never handed over to anybody from the stroke team, even with the appropriate pre-alerts and everything else. That never happened. In fact I never saw a stroke team coming down to a patient while I was there—I never actually handed over to a doctor or a nurse who knew about it. So that’s quite tricky. (P14)

Q7: But then the, sort of, having a bit of an extra hand in the ED, I didn’t really feel all that comfortable with that, really. I felt a bit like I was getting in the way, really, more than anything, what the ED nurse being there, and then the specialist nurse. I felt like I wasn’t really able to add very much, other than getting in the way. (P7)

Q8: Into the main stroke unit at night, and I handed over in the PASTA way. The whole team was stood there so I handed over to a nurse and the doctor at the same time. They just went, ‘That’s a fantastic handover. That’s everything we need to know'. But we have a lot of issues at our local stroke unit, as I say, it isn’t 24 hours, but depending who’s on duty in there they won’t see us until we’ve been triaged by the triage nurse, which if we’re third ambulance in the queue we’re triaged third in the queue and we could be there half an hour before we’re physically getting in and being able to do the PASTA handover to the team, at which point we’ve missed out on everything. (P2)

As with the experiences of prealerting, the participating paramedics found that the receiving hospital teams’ awareness and experience of the PASTA pathway influenced a smooth handover (box 4: Quotation 5). More specifically, their experiences varied depending on whether they were handing over to ED staff or the stroke team directly, as generally, the stroke teams had awareness of the trial and thus were receptive to the enhanced handover. This shared similarities with the paramedics’ experiences of communicating the prealert to the receiving hospital team (box 4: Quotation 6).

Another barrier experienced during the handover occurred in cases where paramedics felt that the hospital team ‘took over’ while they were trying to handover. Paramedics felt that this challenge arose from the professional boundary between the two cultures and expectations of each others’ roles. Stepping outside the role normally expected from the paramedics made some feel outside of their ‘professional comfort zone’ when interacting with hospital staff and they were sometimes apprehensive to ask hospital staff to follow the PASTA handover procedure (box 4: Quotation 7). These experiences of handover were also attributed to general pressures experienced by the health service, because at times the receiving team was simply too busy to follow through the PASTA pathway (box 4: Quotation 8).^11^

### Assisting in hospital and feedback on initial diagnosis by paramedic

Paramedics reported that when they attempted to stay involved with the care of patients following arriving at hospital, they did not feel confident to ask about assisting with activities such as taking the patient to the CT scan. For some, this was because they believed that providing this care was the responsibility of hospital staff (box 5: Quotation 1). Overall, paramedics did not feel that they had a substantive contribution to make in terms of the routine stroke care provided at this stage by hospital staff (box 5: Quotation 2), although personal benefits were noted in terms of continued professional development (box 5: Quotation 3). The experiences recalled shared features with those of the handover, namely, the practicalities of joined up working across the boundary between the two professional cultures was challenging to implement in practice. The difficulties arose due to fact that hospital staff were often not aware or had any prior experience of the PASTA pathway (box 5: Quotation 4). Furthermore, the general pressures of high demand on the ambulance service meant that sometimes paramedic crews were reluctant or unable to stay in hospital for follow-up (box 5: Quotation 5).

Box 5 **Quotations from ‘assisting in hospital and feedback on initial diagnosis by paramedic’**

Q1: No. That’s never happened [helped in hospital]. No, it hasn’t. I have never gone that far. I’ve never needed to —No. Yes. They have got healthcare assistants in there. They have got a stroke nurse. They have got a stroke doctor. It is taken out of my hands pretty much straightaway, especially at this hospital. (P15)

Q2: They're not going to be expecting us to then wait around, go to the scan with them and all that kind of thing. Whether it's just that they don't know about it as well and they aren't expecting that to happen—I didn't say it to them. I probably just didn't feel it appropriate, to be honest and that probably says more about me in terms of not wanting to assume that that would be appropriate to do. It's something worth thinking about though the next time we go in. (P23)

Q3: Again, I've had no issues there. I would say, as a minimum, I've probably stayed 15 min with each patient I've taken in. Partly out of wanting to stay with your patient when you've built up that relationship. Partly because I've wanted to get a bit more experience, because obviously, when someone comes down from the Stroke Unit, they're carrying out their assessment, which overlaps, slightly, with ours. But obviously, they have a wider range of assessment, and I've picked up certain little things. (P6)

Q4: So I wasn't involved with anything; putting a cannula in, going round to CT, none of that happened whilst I was there. She did go to CT but a porter came and took her—So I think it could have gone a lot better but I think it was the stroke team not being aware of what was going on. So they weren't too familiar with it. (P3)

Q5: I would be very happy to wait to know the outcome, but the pressure on ambulance staff is basically so hard that, honestly, they will ask me why I am waiting and I don’t do it, then I think that I try to wait sometimes, this is the reason why I’m trying to be quick in hospital. By waiting in the CT scan of course is satisfying my curiosity, and from the other half it’s not beneficial for the patients because I have provided all the information I know and it’s not beneficial so at expense of ambulance service. (P16)

Q6: Yes. Our controller is aware of the PASTA trial, and obviously we’re going to be delayed further in hospital because with the way the ambulance service go now, they want us to clear as quickly as possible so we’re available for the next job. So, we make our controller aware. We fill in the form and we get feedback, good feedback, from the doctors. We ask them, and we usually get the result of the scan and everything else, which is really good, because it’s always interesting to follow-up a patient anyway. (P4)

Q7: I got feedback on the consultant's initial feelings as to where it was going to go, but no feedback following on from the CT scan, because I was away by then—That everything that was done was right, and our diagnosis, they confirmed that they probably were having a stroke and they were rushing them off to CT, just confirming our diagnosis really, and that we had brought them into the department appropriately. (P17)

When it was possible to complete the follow-up element of the PASTA pathway, the experiences of paramedics were generally positive (box 5: Quotation 6). Paramedics regarded it as a beneficial learning experience professionally because it allowed them to receive feedback and see how the information they provided during handover was used to inform care decisions made by the hospital team (box 5: Quotation 7).^15^ For some, these experiences underpinned reflection on their practice and enriched their professional knowledge. However, the overall view was that time constraints would make it difficult to routinely implement extra time in hospital as a way of contributing towards patient care or professional development.

## Discussion

This qualitative report provides insight into the feasibility and acceptability of an enhanced paramedic role during emergency stroke care and can inform future clinical and research developments. Even though there was overlap with existing practice, participating paramedics valued the content of the PASTA pathway, especially the structure it provided. This was perceived to impact positively on the quality and depth of the information, and the efficiency and smoothness of the assessment/handover process. Furthermore, the enhanced standardisation was perceived to have a positive impact on confidence and professionalism among the paramedics interviewed.

Although formal symptom checklists have been used for some time in emergency stroke care and continue to evolve with the introduction of thrombectomy treatment,^16^ standardisation of information provision during ambulance to hospital handover is not routine practice. The general need to improve handover for all emergency admissions has previously been highlighted by a review of 21 relevant studies, which identified concerns about communication in the chaotic and noisy ED environment, exacerbated by a lack of time and resources.^17^ It was noted that there was no evidence available to show the value of standardisation, even though this was frequently offered as a solution. A review of 12 studies examining structured patient assessment frameworks used by nurses and medical practitioners found improved performance in various documentation and clinical measures,^18^ but also found no examples from prehospital care. More recently, qualitative examination of a generic handover structure ‘IMIST AMBO’ (Identification, Mechanism/medical complaint, Injuries/information relative to the complaint, Signs, Treatment, Allergies, Medications, Background history and Other information) has shown an increase in information conveyance and reduced need for clarifications,^8^ while in a survey of trauma care, handovers observed that the mnemonic ‘ATMIST’ (Age, Time of onset, Medical complaint/injury, Investigation, Signs and Treatment) was the most popular.^19–21^ There have been no previous descriptions of a stroke-specific handover.^22^

Accounts of the handover process highlighted barriers the paramedics experienced when attempting to adhere to a prehospital research protocol, especially due to clinical time and resource pressures during emergency stroke care. This was previously reported in the UK during the RIGHT pilot trial of Glyceryl Trinitrate patches^23^ and by US paramedics who had assisted with the FAST-MAG trial of intravenous magnesium infusion.^24^ In addition, interviewees described the inadvertent guarding of cultural and professional boundaries during their interaction with the hospital team. They noted that the handover was sometimes difficult to initiate, interrupted or taken over by hospital staff. Challenges of this kind showed that implementation of trials is difficult if the hospital team are not informed of, or are not active participants in the intervention.^11^ In the case of PASTA, the research question considered the specific value of an enhanced paramedic role, and so the hospital staff were simply made aware that a stroke assessment trial was taking place in the ambulance service. If the PASTA pathway were to be deployed, then interdisciplinary training may be required to maximise the impact of the handover.

Interviewees also reported that providing practical assistance after handover was often not easy to initiate, as this was not required or not expected by the hospital team. A systematic review of enhanced paramedic roles during and after hospital handover of conditions amenable to time critical therapies (stroke, myocardial infarction and trauma) found no previous studies describing any similar extension of clinical involvement for comparison.^22^ Although interviewees found personal value from staying with the patient longer, it does not seem likely that there was a significant additional contribution to patient care. This is consistent with previous research examining cross-boundary pathways in general, which highlighted the need to consider all aspects of implementation if integration between professions is to succeed.^25^

On completion of the PASTA pathway checklist, paramedics welcomed the opportunity to receive immediate feedback, reflecting their previously recorded enthusiasm for learning more about stroke as an important medical emergency. Previous interviews with Canadian paramedics also found positive perceptions of feedback, but this was described as informal and opportunistic.^15^ Audit of clinical documentation and feedback to individual paramedics has been shown to encourage adherence to standard stroke care assessment but was not in real time and did not allow clarification by the recipient.^7^ Future studies should consider the value of immediate and structured paramedic feedback for those scenarios where specific actions are required during the prehospital assessment.

The strength of this study is in-depth exploration of paramedics’ experiences of a new approach to clinical assessment and handover practices across multiple settings. A limitation of the study is that interviews were conducted with paramedics who volunteered. This may have resulted in only very positive or negative experiences being captured and might not give a full understanding of factors affecting future implementation such as the relevance of different levels of clinical experience or hospital stroke service configuration.

## Conclusion

Paramedics valued the purpose of a complex intervention to enhance the emergency care of stroke patients. Standardisation of the handover process was viewed as the primary benefit of the PASTA pathway. Paramedics found other aspects harder to achieve due to professional boundaries and expectations. Future studies should consider whether interdisciplinary training can improve emergency care for stroke and other common scenarios.
